# Effect of Diets Supplemented With Yeast, Chitin, and Chitosan on the Growth, Immune, and Antioxidant Responses of the Freshwater Prawn *Cryphiops* (*Cryphiops*) *caementarius*

**DOI:** 10.1155/2024/1727130

**Published:** 2024-09-20

**Authors:** Walter Reyes-Avalos, Carlos Azañero-Díaz, Gladis Melgarejo-Velasquez, Carmen Yzásiga-Barrera, Brian Alegre-Calvo, Roberto Lezama-Salazar

**Affiliations:** ^1^ Laboratorio de Acuicultura Ornamental Departamento Académico de Biología Microbiología y Biotecnología Universidad Nacional del Santa, Ancash 02712, Peru; ^2^ Laboratorio de Microbiología y Bioquímica Departamento Académico de Biología Microbiología y Biotecnología Universidad Nacional del Santa, Ancash 02712, Peru; ^3^ Escuela Profesional de Biología en Acuicultura Universidad Nacional del Santa, Ancash 02712, Peru

**Keywords:** acid phosphatase, glutathione-S-transferase, hemocytes, immunostimulants, proPO

## Abstract

The purpose of the present research was to evaluate the effect of diets supplemented with activated yeast, crude chitin, and chitosan on the growth, immune, and antioxidant response of freshwater prawn *Cryphiops* (*Cryphiops*) *caementarius*. Adult male prawns were kept in individual culture vessels installed in aquarium tanks. The basal diet (control) was supplemented with activated *Saccharomyces cerevisiae* yeast (60 g/kg), crude chitin (20 g/kg), and chitosan (1 g/kg). Each dietary treatment consisted of three replicates. The diet supplemented with activated yeast causes greater growth, as well as a greater number of total hemocytes (82.54 × 10^5^ cells/mL), semigranular (59 × 10^5^ cells/mL), and granular (18.67 × 10^5^ cells/mL) hemocytes and without atypical hemocytes. Furthermore, a higher number of hemocytes positive for prophenoloxidase (98%), a shorter hemolymph clotting time (42.87 s), a higher activity of acid phosphatase (12.50 U/mL) and glutathione-S-transferase (GST) (0.186 U/mL) were also observed in dietary yeast group. On the contrary, there were no differences in the activity of superoxide dismutase (SOD) enzyme in prawns from any dietary treatment. Results from this research demonstrate for the first time that the diet containing activated yeast increases the growth and immune response of the freshwater prawn through a significant increase in hemocyte and acid phosphatase levels, a decrease in hemolymph clotting time, and a greater number of proPO-positive hemocytes. However, activated yeast is not effective in increasing the activity of antioxidant enzymes SOD and GST. Therefore, the activated yeast diet can be useful to improve the aquaculture production of *C*. (*C*.) *caementarius* and possibly of other commercially important crustaceans.

## 1. Introduction

In crustaceans, the nonspecific or innate immune response is the first line of defense against pathogens, and immunostimulants promote growth with high survival and achieve greater resistance to diseases [1] and environmental stress [2–4]. Once harmful agents reach the hemocoel, they must contend with coordinated efforts for cellular and humoral immunity [5]. Shrimp hemocytes have the function of resistance to pathogens, through a variety of cellular and humoral responses that include clotting and lysis of the pathogen, wound healing, and among others [6]. In the aquaculture industry, there are several immunostimulant agents commonly used to increase the immune response of several important species [7]. The use of immunostimulants in the diet of aquaculture species is also a strategy to avoid the use of antibiotics [8, 9], since it has been reported that bacteria/pathogens resistant to antimicrobials are often transferred from aquatic organisms to humans through the food chain [1].

Yeast (*Saccharomyces cerevisiae*) is a probiotic that is used in diets to provide immunity against pathogens [10] in both fish and crustaceans [11]. It has been demonstrated that yeast diet favors growth, intestinal microbiota, intestinal morphology, and the immune response in shrimp, prawn, and crayfish species [12–14]. For instance, in the whiteleg shrimp *Penaeus vannamei*, yeast increases the productive yield and activities of the antioxidant enzymes superoxide dismutase (SOD) and catalase (CAT) [12].

Chitin is the most abundant biopolymer on the planet after cellulose, which is present in the cuticle of the exoskeleton of arthropods and insects [15, 16]. Chitin is used in diets to increase the growth and immunity of fish and crustaceans [9], resulting in increased hemocytes and prophenoloxidase (proPO) activities of *Procambarus clarkii* [17] and *Macrobrachium rosenbergii* [18], while in *P. vannamei*, it promotes cellular respiratory burst (RB), phagocytic activity (PA) of hemocytes and in SOD, as well as in the expression of genes related to the immune system [8].

Chitosan, derived from the deacetylation of chitin with an alkali [19], is an edible, biocompatible, biodegradable, nontoxic, and safe natural polymeric material [20] that has an immunostimulant function in fish and crustaceans of interest in aquaculture [8, 9, 21]. It has been shown that dietary chitosan diets stimulate hemocyte proliferation, and proPO and SOD activity in adult individuals of *P. clarkii* [17], whereas in *P. monodon*, it contributes to the assimilation of antioxidants from foods to improve antioxidant activities and the ability to resist stress generated by low oxygen content in the environment [2]. In *P. vannamei*, dietary chitosan positively regulates several immune responses, including RB, PA, and SOD activity [8], and restores glutathione-S-transferase (GST) and SOD activity, while decreasing lipid peroxidation in the gills and increasing reduced glutathione levels in the hepatopancreas [3]. Furthermore, chitosan injection causes greater immune capacity by increasing hemocytes, phenoloxidase (PO) activity, and RB in *P. vannamei* [22].

The prawn *Cryphiops* (*Cryphiops*) *caementarius* is a palaemonid of economic and nutritional importance in Peru and Chile, whose extraction has been carried out for more than 60 years in the rivers with the highest population density, such as the Cañete, Mala, Majes-Camaná, Ocoña, Pativilca, and Tambo rivers in Peru [23–26]. Fluctuations in population density were observed across this period, most likely due to intense extractive activity and other anthropogenic activities such as the use of agricultural pollutants and ecosystem engineering [27], making it necessary to establish the conditions for its culture in captivity.

The culture of *C*. (*C*.) *caementarius* is still in its early stages and only practiced at the family level in some valleys along the Peruvian coast and thereby there is a need for the development of a balanced diet that improves growth performance and to strengthen the immune system with antioxidants. Despite the aquaculture importance of *C*. (*C*.) *caementarius*, studies on its nutrition and growth performance are still limited [28–31]. Previous works on *C*. (*C*.) *caementarius* suggested that activated yeast from *S. cerevisiae* and crude chitin supplemented in diets promote hemocyte proliferation [16, 32, 33], while dietary chitosan increases the proportion of proPO-positive hemocytes [34]. However, to our knowledge, no study has yet analyzed the effects of their antioxidant response in the freshwater prawn. Therefore, the objective of this research was to evaluate the effect of diets supplemented with activated yeast, crude chitin, and chitosan on the growth, immune, and antioxidant response of the freshwater prawn *C*. (*C*.) *caementarius*.

## 2. Materials and Methods

### 2.1. Obtaining Activated Yeast

Instant dry sweet dough yeast (*S. cerevisiae*) (Mauripan) was purchased from a local store and was activated as follows [33]: 75 g of yeast was added to 500 mL of sugar water (4% of the volume of water at 40°C), then incubated in an oven (37°C for 24 h), and then centrifuged (5000 rpm for 15 min). The supernatant was discarded, and the precipitate was used as activated yeast.

### 2.2. Obtaining Crude Chitin

The crude chitin flour was obtained from prawn exoskeletons (cephalothorax and chelipeds) that were used in a previous study carried out by our research team [16], the approximate analysis is shown in Table 1.

### 2.3. Obtaining Chitosan

The commercial chitosan was of low molecular weight (76% degree of deacetylation, Sigma–Aldrich, Cat. 448869).

### 2.4. Diet Preparation

The diets were prepared using the formulation, shown in Table 2, following the procedure described in our previous research [16], with some modifications. Briefly, once the wet mixture of all ingredients indicated in the basal diet was obtained, pellets (3 mm Ø) were obtained with a manual press, then they were dried in an oven (40°C for 24 h) and stored in airtight bags until used. Then, each experimental diet was prepared separately by grinding dry pellets of the basal diet (control diet), following the dietary supplementation indicated in Table 2. Water (20%) was then added to each mixture to homogenize and obtain pellets using the same procedure as that of the basal diet. The pellets were stored in airtight bags until use.

### 2.5. Capture, Transport, and Acclimatization of the Prawns

Prawns (*n* = 100) were caught from the Pativilca River (Lima, Peru) with the help of local fishermen. Each prawn was placed in a sieved plastic cup (200 mL) for transport according to the technique described by Reyes-Avalos [35] and at the density of 50 prawns/plastic box (0.60 × 0.40 × 0.35 m, 45 L). The land transportation time lasted 4 h. In the laboratory, the prawns were acclimated for 8 days in the same transport vessels but distributed within three fiberglass tanks (0.66 × 0.44 × 0.46 m, 120 L) and fed the basal diet. In addition, a 30% water change was conducted every 2 days, including the removal of food remains and prawns' feces. The experiment procedure with live animals followed the current Peruvian law (Law 30407, Animal Protection and Welfare Law).

### 2.6. Cultivation System

The culture system was the same as previously reported elsewhere [35] and consisted of 12 aquariums (0.60 × 0.40 × 0.45 m, 90 L), each housing six individual culture containers (201 cm^2^ and 6 cm high) arranged in two groups of three levels.

### 2.7. Selection, Stocking, and Feeding

Prawns of the species *C*. (*C*.) *caementarius* were identified using a taxonomic key [36]. We selected only male prawns because of the pronounced sexual size dimorphism in prawn species, where males grow larger than females [23]. At present, aquaculture production of this species is still in the experimental stage. However, future commercial operations using all-male populations are expected to reach market weights in shorter rearing periods [35, 37]. The sex of males was verified by the presence of a genital pore in each coxopodite of the fifth pair of periopods. Male prawns (*n* = 72) of similar size (5.96 ± 0.24 cm total length and 8.63 ± 0.85 g total weight) were selected if they had complete cephalothoracic appendages and no signs of lacerations on the body. The selected prawns were randomly assigned one to each culture container (six prawns/aquarium = 25 prawn/m^2^). The feeding rate was 5% of wet weight per day and the feeding frequency was twice a day (40% at 08 : 00 h and 60% at 18 : 00 h) for 30 days to evaluate the immediate effect of the used stimuli [13, 14].

### 2.8. Growth and Survival

Total weight (g) was recorded on an Adam AQT600 digital balance (±0.1 g), and the weight gained and survival were calculated using the following formulae:

(1) $$\begin{array}{c} \text{Weight gained }\left(\%\right)=\left[\left(\text{Final weight}-\text{initial weight}\right)/\text{ Initial weight}\right]\times 100, \end{array}$$

(2) $$\begin{array}{c} \text{Survival rate }\left(\%\right)=\left(\text{Final number prawn}/\text{Initial number prawn}\right)\times 100. \end{array}$$

### 2.9. Immune Response

At the end of the experiment, six prawns from each treatment were chosen in the C or Do molting stages [38] to extract hemolymph only once according to the method previously described for the species [16]. Hemolymph was extracted from each prawn only once. A drop of hemolymph was placed in the Neubauer chamber and hemocyte counting was performed in four quadrants ( = 0.1 mm^3^) under a LEICA DM LS2 conventional light field microscope provided with phase contrast. The types of hemocytes were granular (G), semigranular (SG), hyaline (H), and atypical (A) [39]. The total (THC) and differential (DHC) count of hemocytes was calculated with the following formula [40]:

(3) $$\begin{array}{c} \text{THC }\mathrm{or}\text{ DHC }\left(\text{cells}/\text{mL}\right)=\left(\text{Counted cells}\times 5\times 1000\right)/0.4. \end{array}$$

The clotting time of prawn hemolymph was determined in capillaries without heparin (1.1–1.2 mm inner Ø, 75 mm length) [41]. The proPO was determined in the hemocytes of prawn (*n* = 2 per treatment) in C or Do molt stages [38] and for this purpose, 100 randomly selected hemocytes were counted on the slide, and the stained hemocytes in black were considered proPO-positive [18]. Acid phosphatase (ACP) activity in prawn plasma was determined with a commercial test kit (Sigma–Aldrich, Cat. MAK446) following the company's protocol.

### 2.10. Antioxidant Response

The activities of SOD (in plasma and in lysed hemocyte solution) and GST (in plasma) of prawns were determined with commercial test kits (Sigma–Aldrich, Cat. CS0009, and MAK453, respectively) following each company's protocol.

### 2.11. Water Quality

The temperature was recorded with a digital thermometer (±0.1°C), the dissolved oxygen and the percentage of saturation with a Hanna HI 9146 Oximeter (±0.01 mg/L), the pH with a PH-222 digital pH meter (±0.02 units), which were measured twice a week. Total ammonia nitrogen (TAN = NH_3_-N + NH_4_-N), ammonia nitrogen (NH_3_-N), ammonium nitrogen (NH_4_-N), nitrite nitrogen (NO_2_-N), and nitrate nitrogen (NO_3_-N) were measured once a week with a commercial kit (Hanna), whose reading was carried out with the Hanna multiparameter photometer HI 83399 (±0.01 mg/L).

### 2.12. Analysis of Data

The normality of the data was analyzed with Shapiro–Wilk and Levene's test. Differences among treatment means were determined at 95% by one-way analysis of variance (ANOVA) and Duncan׳s post hoc test. Statistical analyses were performed on SPSS software version 25 for Windows.

## 3. Results

### 3.1. Growth and Survival

The weight gained during the experimental period was greater (*p* < 0.05) in prawns fed activated yeast (10.60% ± 0.08%) and in the control (9.80% ± 1.13%), in relation to those with the crude chitin (7.21% ± 1.53%) and chitosan (6.77% ± 2.18%). Survival was similar (*p* > 0.05) among treatments, being 77.77% ± 9.62% in prawns fed activated yeast and chitin diets, 83.33% ± 0.00% with chitosan, and 88.86% ± 9.62% with the control diet. Prawn mortality was due to unknown causes.

### 3.2. Immune Response

**Figure 1:**
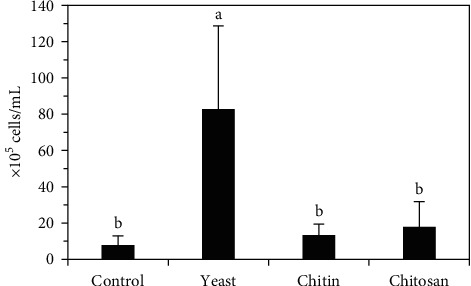
Concentration of total hemocytes of *C*. (*C*.) *caementarius* fed with diets supplemented with activated yeast, crude chitin, and chitosan. Columns with different letters indicate significant differences (*p* < 0.05).

**Figure 2:**
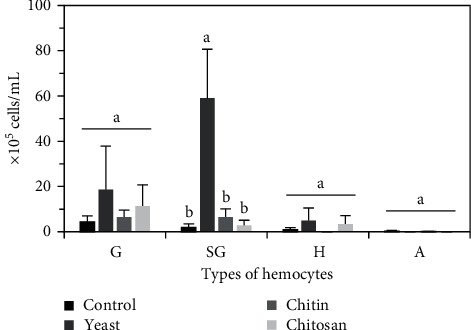
Concentration of granular (G), semigranular (SG), hyaline (H), and atypical (A) hemocytes of *C*. (*C*.) *caementarius* fed with diets supplemented with activated yeast, crude chitin, and chitosan. Columns with different letters indicate significant differences (*p* < 0.05).

Our results showed that the prawns fed activated yeast had a higher number of total hemocytes (82.54 × 10^5^ cells/mL) (*p* < 0.05), than those fed chitosan (17.63 × 10^5^ cells/mL), crude chitin (13.04 × 10^5^ cells/mL), and the control (7.58 × 10^5^ cells/mL) (Figure 1). We also observed that the diet with activated yeast caused a greater number of SG hemocytes (59.00 × 10^5^ cells/mL), which represent 71% of the total hemocytes, followed by G hemocytes (18.67 × 10^5^ cells/mL), which represent 23%, H hemocytes (4.88 × 10^5^ cell/mL), which represent 6% of the total hemocytes. On the other hand, prawns fed dietary chitosan maintained high values of G hemocytes (11.33 × 10^5^ cells/mL) and a low proportion of SG hemocytes (2.83 × 10^5^ cells/mL), H hemocytes (3.46 × 10^5^ cells/mL), and no atypical hemocytes were observed (Figure 2).

**Figure 3:**
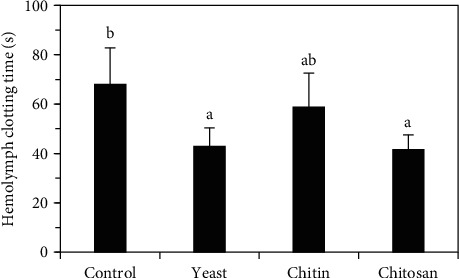
Hemolymph clotting time (s) of *C*. (*C*.) *caementarius* fed with diets supplemented with activated yeast, crude chitin, and chitosan. Columns with different letters indicate significant differences (*p* < 0.05).

The clotting time of prawns fed activated yeast and chitosan was the lowest, and significantly lower than that of the control group (*p* < 0.05), but there was no significant with those fed chitin (*p* > 0.05) (Figure 3).

**Figure 4:**
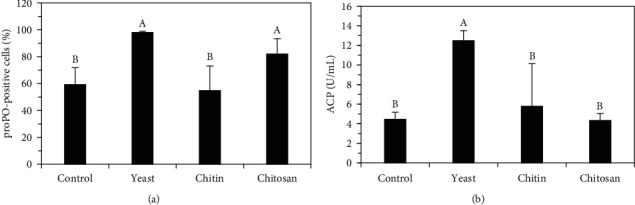
(a) Prophenoloxidase (proPO)-positive hemocytes and (b) acid phosphatase (ACP) activity in plasma of *C*. (*C*.) *caementarius* fed with diets supplemented with activated yeast, crude chitin, and chitosan. Columns with different letters indicate significant differences (*p* < 0.05).

Figure 4 shows the values of the analyzed immunological enzymes, where prawns fed activated yeast and chitosan had a greater number (*p* < 0.05) of proPO-positive hemocytes in their hemolymph (98% and 82%, respectively) than with crude chitin and the control treatments (54.81% and 59.20%, respectively). ACP was significantly higher (*p* < 0.05) in prawns fed activated yeast (12.50 U/mL) than those fed crude chitin (5.78 U/mL), chitosan (4.33 U/mL), and the control diet (4.44 U/mL).

### 3.3. Antioxidant Response

**Figure 5:**
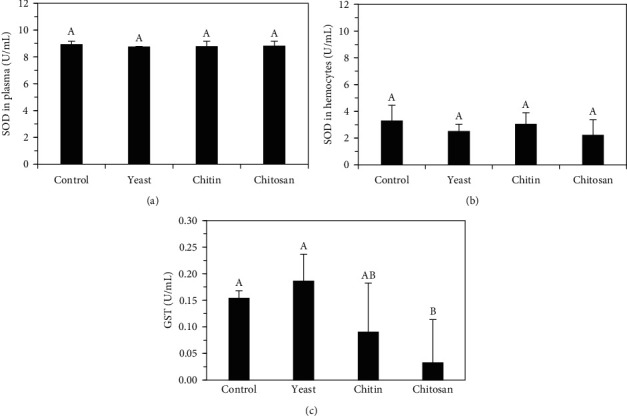
(a) Activity of superoxide dismutase (SOD) in plasma, (b) in solution of lysed hemocytes, and (c) glutathione-S-transferase (GST) activity in plasma of *C*. (*C*.) *caementarius* fed with diets supplemented with activated yeast, crude chitin, and chitosan. Columns with different letters indicate significant differences (*p* < 0.05).

The values of the activity of antioxidant enzymes obtained in each dietary treatment are shown in Figure 5. The SOD in the plasma (Figure 5a) and in the hemocytes (Figure 5b) of the prawns were similar (*p* > 0.05) among dietary treatments whose average values were 8.81 ± 0.26 U/mL and 2.76 ± 0.93 U/mL, respectively. However, greater SOD activity in hemocytes was observed in the control treatment (plasma: 8.93 U/mL, hemocytes: 3.29 U/mL). GST (Figure 5c) was high and similar (*p* > 0.05) in prawns fed activated yeast (0.186 ± 0.050 U/mL), control diet (0.154 ± 0.014 U/mL), and crude chitin (0.090 ± 0.092 U/mL), while the lowest value was observed in the chitosan treatment (0.032 ± 0.081 U/mL).

### 3.4. Water Quality

The water quality parameters were similar among dietary treatments (Table 3); however, the nitrogen parameters were higher (*p* < 0.05) only in the control treatment, except for the nitrate concentration.

## 4. Discussion

### 4.1. Growth and Survival

The present research revealed for the first time that the diet containing activated yeast causes greater weight gain in male individuals of *C*. (*C*.) *caementarius*, in relation to the diets treated with crude chitin and chitosan. These results suggest that the nutritional value of the yeast [42] improves the immune and antioxidant response, even in short-term culture periods (30-day experiment). Similar growth performance results were obtained in other related species fed yeast-containing diets. In *P. clarkii*, growth-promoting effects of dietary yeast were observed after 28 days [13], while in *P. vannamei*, feeding for 60 days with a yeast-containing diet resulted in the best growth performance, which could be attributed to the valuable nutrients [12]. On the other hand, our results also showed that lower growth rates were obtained with the crude chitin and chitosan diet treatments, which are in agreement with those of previous crustacean studies that reported relatively low growth rates in the same prawn species [16, 34] and in *P. monodon* [43] fed crude chitin and chitosan diets. However, contrasting results were reported in *P. vannamei* where dietary chitosan promoted growth [44]. The reason why chitin and chitosan in diets generate opposite results in crustaceans is still not well-known [2]. It has been suggested however that dietary chitosan interferes with the assimilation of proteins and lipids in *P. monodon* [43]. The digestion of chitin depends on the presence of chitinases [45], and whose enzymes are essential for the digestion and molting of *M. nipponense* [46]. Low growth rates of *M. tenellum* have also been associated with a decrease in chitinase activity [47].

Our data revealed that none of the experimental diets significantly affected (*p* > 0.05) the survival rate of *C*. (*C*.) *caementarius*, which ranged from 77% to 88%, and mortality was due to unknown causes. Dietary yeast, chitin, and chitosan have been reported to result in high survival rates in prawn species. For instance, Cornejo et al. [32] reported 0% mortality by using dietary yeast in *C*. (*C*.) *caementarius*. Later, Reyes-Avalos et al. [34] showed that the same prawn species fed crude chitin (20 g/kg) resulted in 94% survival. The same study demonstrated that dietary chitosan (1 g/kg) resulted in 55% survival, but mortality was due to molting. Survival rates in *M. tenellum* fed 15%–20% crude chitin were 72.2%–83.3%, respectively [47].

### 4.2. Immune Response

The prawn innate immune system comprises cellular and humoral components [6]. The total hemocyte count is related to various factors and some extent reflects the immune response and health status of the host [48]. In the present research, male *C*. (*C*.) *caementarius* prawns fed activated yeast showed the highest total hemocyte count (82.54 × 10^5^ cells/mL, *p* < 0.05, Figure 1), which were from 5-fold to 11-fold higher than those of the prawns fed chitosan (17.63 × 10^5^ cells/mL), crude chitin (13.04 × 10^5^ cells/mL), and control (7.58 × 10^5^ cells/mL) treatments, demonstrating the efficiency of yeast in the hemocyte proliferation and immune-enhancing activity in this prawn species. These results corroborate previous studies that reported the proliferation of total hemocytes in the same prawn species fed activated yeast (60 g/kg) [33], and in *P. vannamei* fed yeast hydrolysate (10 g/kg) [49].

Crude chitin and chitosan are immunostimulants known to increase the count of circulating hemocytes in crustaceans [17, 22]; however, in the present research, we did not observe the proliferation of hemocytes in *C*. (*C*.) *caementarius* fed with those agents. In the case of crude chitin, the nutrient content likely affected the assimilation of chitin and thus the immunological effect, which was also evident with proPO. In certain crustaceans, excess of pure chitin (10%) in the diet affects nutrient absorption [50] due to the difficulty in metabolizing excess glucosamine [47]. Our approximate composition analysis results of crude chitin showed a 5.85% pure chitin (Table 1). However, a higher level of chitin content is expected to be present in the crude chitin treatment because of the extra chitin content from the fish meal (10.38%) [51]. Furthermore, the interaction of dietary ingredients has been proposed to affect the efficiency of food additives [52]. In this sense, the crude chitin used also has a high carbohydrate and protein content, thereby these nutrients likely affected the immunostimulant effect of chitin, since it is known that in other related crustaceans such as *M. acanthurus* an excess of proteins and carbohydrates in their diet causes an inflammatory reaction [53]. On the other hand, the lack of proliferation of hemocytes in prawns fed dietary chitosan observed herein was likely due to the concentrations of nitrogen products in the culture water that attenuated the immunostimulant effect of dietary chitosan. It was previously reported that in *M. amazonicum* and *P. vannamei*, a decrease in their hemocyte count occurs after being exposed to high concentrations of ammonia in water [54, 55]. In this light, it is advisable to further investigate the effects of nitrogen products in the culture water of prawn species to establish their dangerous concentration limits.

We also observed that the activated yeast used in the diet of *C*. (*C*.) *caementarius* increases SG and G hemocytes, which represent 71% and 23% of the total hemocytes, respectively, (Figure 2), which is within the range reported for other crustaceans [6]. Furthermore, the concentrations of SG and G hemocytes obtained in prawns fed yeast (59.00 × 10^5^ cells/mL and 18.67 × 10^5^ cells/mL, respectively) were from 2-fold to 20-fold times higher (*p* < 0.05) than those observed in the crude chitin (6.54 × 10^5^ cells/mL and 6.46 × 10^5^ cells/mL, respectively) and chitosan (2.83 × 10^5^ cells/mL and 11.33 × 10^5^ cells/mL, respectively) treatments. Similar value results were reported for the same prawn species fed activated yeast [32, 33] and crude chitin [16].

In crustaceans, granular hemocytes are unstable cells that degranulate and release their contents during the clotting process [56]. Clotting in crabs and shrimps is mediated by the polymerization of plasma proteins, catalyzed by transglutaminase in the presence of calcium ions [6]. Invading bacteria and fungi become trapped within clots to prevent septicemia and mycosis [5]. In the present study, the hemolymph clotting times of *C*. (*C*.) *caementarius* fed activated yeast (42.87 s) and chitosan (41.59 s) were significantly (*p* < 0.05) shorter than that of the control diet (67.98 s), although clotting time of the control diet was closer to that of the crude chitin group (58.73 s). These results are supported by a previous study by Reyes-Avalos et al. [16] where a high clotting time (70.90 s) was obtained in freshwater prawn individuals fed 20% crude chitin. In other crustaceans, such as *M. rosenbergii*, it was demonstrated that the hemolymph clotting time can be reduced from 60 to 45 s by an increase in hemocyte transglutaminase levels obtained with dietary *Ganoderma lucidum* polysaccharides (GLP) [57]. Herein, the shorter clotting time observed in *C*. (*C*.) *caementarius* is related to the greater number of proPO-positive hemocytes obtained in the prawns fed mainly with activated yeast and chitosan, suggesting that clotting was triggered by some component present in the proPO system, as it was previously demonstrated in *P. virginalis*, where proPO components were observed to induce hemocyte degranulation [56].

Our results are the first to show that dietary active dry yeast causes a high proportion of proPO-positive hemocytes and high ACP activity in *C*. (*C*.) *caementarius*, which led to the increase of the immune response in this prawn species. In *P. vannamei*, the cultured yeast diet modulates PO, which induces antimicrobial substance to improve phagocytosis in shrimp hemocytes, and ACP, which hydrolyzes phosphate conjugates in many organic compounds [12]. Furthermore, our results also suggest that *C*. (*C*.) *caementarius* has a greater affinity for *β*-glucans that must come from the digestion of yeast cells in the animal's digestive tract. Yeast cell walls contain glucans as immunostimulants [58], which increase circulating hemocytes, phagocytosis, encapsulation, ProPO activity, and melanization [42]. In various crustaceans, there are *β*-glucan recognition proteins that are involved in the activation of proPO of these organisms, suggesting that they interact with the proPO system [59]. For example, in *P. clarkii* fed *S. cerevisiae*, the significant increase in proPO gene expression in hepatopancreas reflects the abundance of *β*-glucans in the yeast cell wall [13], whereas the modulation of ACP and PO activities in *P. vannamei* before and after being exposed to a pathogenic bacterium indicates a good immune response because the *β*-glucan of yeast cells contains binding receptors that undulate on the cell surface to phagocytose pathogens [12].

The proPO system, a widely recognized innate immune system in crustaceans, participates in hematopoiesis [60]. We found that *C*. (*C*.) *caementarius* fed with the diet with activated yeast increased (*p* < 0.05) proPO-positive cells (98%), as well as SG and G hemocytes, resulting in the improvement of the prawn's immune system. SG hemocytes, which were the most abundant hemocytes found in the freshwater prawns fed with the yeast diet, contain small eosinophilic granules that are responsible for the recognition of microorganisms leading to encapsulation, coagulation, and occasional phagocytosis [6]. Studies in other related crustaceans like *M. rosenbergii* demonstrated that PO exists in all three types of hemocytes, but greater activity is present in G and SG hemocytes, which is why they have strong immunocompetence [61]. The proPO of granular hemocytes participates in melanization during the encapsulation of microorganisms and is one of the vital components of the immune system of shrimps [6].

We observed a low proportion of proPO-positive hemocytes in *C*. (*C*.) *caementarius* fed crude chitin (54.81%), which was similar to those fed with the control treatment (59.20%). Contrastingly, higher levels of proPO-positive hemocytes (98%) were found in those individuals from the activated yeast diet group, which suggests that the high nutrient content present in the crude chitin and control diets [16] might have affected the immune response due to not having an adequate balance of nutrients. In *M. acanthurus*, protein and carbohydrates excess in the diet can cause excessive production of free radicals that induce an inflammatory reaction mediated by proPO and PO [53]. Nevertheless, contrasting results were observed in *M. rosenbergii* fed dietary chitin, due to the presence of a protein in the hemolymph that recognizes chitin, which increases proPO activity (70.4%) in hemocytes [18]. On the other hand, there was a higher proportion of proPO-positive hemocytes (82%) observed in the *C*. (*C*.) *caementarius* belonging to the chitosan treatment group, which was even higher than previously reported proPO activity results (66.28%) in the same prawn species subjected to the same chitosan dietary treatment [34]. Similarly, greater proPO activity in plasma was obtained in *P. clarkii* fed chitosan [17], while in *P. monodon*, it was reported that chitosan has a superior immunostimulant effect than dietary chitin [2].

### 4.3. Antioxidant Response

SOD activity in the plasma of *C*. (*C*.) *caementarius* was similar (*p* > 0.05) among the dietary treatments evaluated herein. In *P. clarkii*, the diet with chitosan causes higher SOD activity in the animal's plasma than dietary chitin [17]. In *P. vannamei*, SOD activity in plasma was found to be very high in those individuals fed cultured yeast (*S. cerevisiae*) due to the presence of metabolic products [12]; although, with diets containing yeast hydrolysate there was lower SOD activity [62]. On the other hand, the SOD activity in the plasma of *C*. (*C*.) *caementarius* (Figure 5a) was three times higher than what was observed in the hemocytes (Figure 5b), where the diets with yeast and chitosan caused lower SOD activity, in relation to crude chitin and the control treatments, although without significant differences (*p* > 0.05) (Figure 5b). The reduction in SOD activity observed in hemocytes of the freshwater prawn may be associated with a decrease in oxidative stress and free radicals [2]. In *P. vannamei*, diets with chitin and chitosan had a positive effect on modulating the immune response including SOD activity in hemocytes, but chitosan regulates the reactive oxygen system by scavenging free radicals, thus preventing damage to the organism [8]. The greater SOD activity in the hemocytes of *C*. (*C*.) *caementarius* was observed in those of the control group (Figure 5b) in relation to the other dietary treatments, although without significant differences (*p* > 0.05). This could be due because of the higher levels of nitrogen products detected in the culture water from the control group, which was twice the concentration of the other treatments (Table 3). In *M. rosenbergii* exposed to ammonium stress, an increase in SOD occurs in response to counteracting free radicals [63]. SOD is in the cytosol and mitochondria [64] and is the first detoxification enzyme and the most powerful antioxidant in the cell, acting as a component of the first-line defense system against reactive species of oxygen [65]. Interestingly, the exposure of the prawns to challenging situations might increase the advantageous immunological effects of dietary yeast, since it has been reported that the effects of dietary yeast products on immune responses become more noticeable when shrimps face pathogenic challenges or stressors [49].

The GST activity of crustaceans is generally evident in the gills and hepatopancreas because they have the highest metabolic rates due to their direct interaction with the environment [66]. GST participates in defense against xenobiotic-induced oxidative stress [67]. Our experiments also demonstrated for the first time that diets containing activated yeast, crude chitin, and even the control diet increase (*p* < 0.05) GST activity in *C*. (*C*.) *caementarius* plasma (Figure 5c), suggesting that these immunostimulant agents could protect animals from external stressors, as reported to occur in other crustacean species with high levels of GST activity, such as *Eriocheir sinensis* [4] and *P. clarkii* [68]. On the other hand, we can deduce from our results that our chitosan-containing diet is not effective to protect *C*. (*C*.) *caementarius* against environmental stress. A possible explanation for that result might be that higher chitosan concentrations are needed to stimulate the GST activity in this prawn species. However, a previous study reported that a diet with 1 g/kg chitosan was effective to successfully restoring the activity of GST in *P. vannamei* [3]. This evidence stresses the importance of further research using different levels of chitosan-containing diets to assess the antioxidant efficacy of this agent in *C*. (*C*.) *caementarius*.

In addition, it has been demonstrated that GST increases after crustaceans are exposed to ammonia stress [69]. This would explain the higher GST activity in the prawns belonging to the control treatment, in relation to those from the crude chitin and chitosan groups, as there was a higher concentration of nitrogen products in the culture water of this treatment, where we observed undigested pellets (data not presented). The low hydrostability (76%) of this control diet, which was previously reported by Reyes-Avalos et al. [16], might be the reason for the observed residual pellets in the culture water of our control system. The other environmental parameters of the culture water used in our supplemented diet treatments (Table 3) were similar to those of what was previously reported for the natural environment of the freshwater prawn [25] and controlled culture systems [16, 31].

## 5. Conclusions

This research demonstrated the great potential of activated yeast (*S. cerevisiae*) supplemented in the freshwater prawn diet (60 g/kg), which causes greater growth and positive immune response in male individuals of this high-value species. However, it does not greatly enhance the activity of antioxidant enzymes compared to the results obtained with the chitin treatment and control diet. Further studies are needed to evaluate the capacity of dietary yeast to stimulate biochemical protection mechanisms in prawns against environmental and nutritional stress that often occurs under aquaculture conditions.

## Figures and Tables

**Table 1:** Proximate composition of crude chitin (dry basis).

Composition	Percentage
Moisture	9.20 ± 0.80
Crude protein	33.70 ± 0.20
Crude fat	8.20 ± 0.50
Crude fiber	2.60 ± 0.10
Ash	9.90 ± 0.10
Chitin	5.85 ± 0.64
Carbohydrates	45.60 ± 0.20
Carbohydrates/fats	10.00 ± 0.50

*Note:* Data are expressed as mean ± standard deviation.

**Table 2:** Ingredients of basal diet composition, dietary supplementation, and proximal composition of control and experimental diets (dry basis).

Parameters	Diets			
	Control	Yeast	Chitin	Chitosan
Ingredients of diet (g/kg)				
Fish meal	300.00	300.00	300.00	300.00
Soybean meal	210.00	210.00	210.00	210.00
Corn flour	154.50	154.50	154.50	154.50
Paprika flour	2.50	2.50	2.50	2.50
Fish oil	20.00	20.00	20.00	20.00
Soybean oil	5.00	5.00	5.00	5.00
Corn oil	5.00	5.00	5.00	5.00
Soy lecithin^a^	10.00	10.00	10.00	10.00
Rice powder	200.00	200.00	200.00	200.00
Molasses	27.00	27.00	27.00	27.00
Zeolite	40.00	40.00	40.00	40.00
Common salt	20.00	20.00	20.00	20.00
Vitamins and minerals^b^	3.00	3.00	3.00	3.00
Butylated hydroxytoluene	1.00	1.00	1.00	1.00
Sorbic acid	2.00	2.00	2.00	2.00
Dietary supplementation (g)				
Basal diet	1000.00^c^	940.00	980.00	999.00
Activated yeast	0	60.00^d^	0	0
Crude chitin	0	0	20.00^c^	0
Chitosan	0	0	0	1.00^e^
Proximal composition (%)				
Crude protein	29.15 ± 0.21	32.00	34.55 ± 0.21	35.18 ± 0.15
Crude fat	5.58 ± 0.53	8.10	8.07 ± 0.54	7.12 ± 0.30
Crude fiber	1.80 ± 0.14	2.60	2.20 ± 0.14	2.40 ± 0.15

^a^Purified soy lecithin (1200 mg soft capsules with phosphatides ≥250 mg).

^b^Each 100 g contains vitamin B1 (thiamine) 500 mg, vitamin B2 (riboflavin) 1200 mg, vitamin B6 (pyridoxine) 900 mg, vitamin B12 (cyanocobalamin) 1000 µg, biotin 2 mg, nicotinamide 2000 mg, calcium pantothenate 1000 mg, sodium chloride 2000 mg, potassium chloride 8000 mg, and magnesium sulfate 1200 mg.

Dietary supplementation and proximal composition were obtained from previous related research:

^c^Reyes-Avalos et al. [16].

^d^Cornejo et al. [32].

^e^Reyes-Avalos et al. [34].

**Table 3:** Culture water quality parameters of *C*. (*C*.) *caementarius* fed with diets supplemented with activated yeast, crude chitin, and chitosan.

Parameters	Diets			
	Control	Yeast	Chitin	Chitosan
Temperature (°C)	21.56 ± 0.34^a^	21.70 ± 0.07^a^	21.75 ± 0.16^a^	21.75 ± 0.11^a^
Dissolved oxygen (mg/L)	6.28 ± 0.29^a^	6.22 ± 0.28^a^	6.28 ± 0.14^a^	6.36 ± 0.26^a^
Oxygen saturation (%)	70.73 ± 0.26^a^	69.75 ± 1.17^a^	70.83 ± 1.44^a^	72.60 ± 3.03^a^
pH (units)	7.96 ± 0.07 ^a^	7.96 ± 0.17^a^	7.98 ± 0.08^a^	7.93 ± 0.07^a^
Total ammoniacal nitrogen (mg/L)	1.13 ± 0.02^b^	0.50 ± 0.03^a^	0.51 ± 0.22^a^	0.60 ± 0.03^a^
Ammonia nitrogen (mg/L)	0.55 ± 0.01^b^	0.24 ± 0.01^a^	0.25 ± 0.11^a^	0.29 ± 0.01^a^
Ammonium nitrogen (mg/L)	0.58 ± 0.01^b^	0.26 ± 0.01^a^	0.26 ± 0.11^a^	0.31 ± 0.01^a^
Nitrite nitrogen (mg/L)	0.50 ± 0.01^b^	0.00 ± 0.00^a^	0.00 ± 0.00^a^	0.25 ± 0.35^a^
Nitrate nitrogen (mg/L)	6.10 ± 2.26^a^	5.30 ± 1.27^a^	5.40 ± 1.70^a^	5.45 ± 0.64^a^

*Note:* Data are expressed as mean ± standard deviation. Data with different superscript letters in the same row indicates significant differences (*p*  < 0.05).

## Data Availability

All data are presented in this research article.
